# Two Cases of Scurvy Attended with Some Uncommon Circumstances

**Published:** 1781-08

**Authors:** 


### SECTION II.

### Essays and Observations.

*Two cases of ſcurvy attended with ſome uncommon circumſtances. By Mr.* William Coleman, *ſurgeon at Sandwich in Kent. Communicated by* Samuel Foart Simmons, M. D. F. R. S. *Read* July 9, 1781.

MISS M. C. aged about 17 years, of a healthy, ſtrong conſtitution, took cold while on a viſit at a neighbouring village, and on her return to Sandwich in the evening of Monday, May 1, complained of being ſlightly indiſpoſed. The next morning ſhe told me ſhe had ſpit blood from her gums, and I adviſed her to waſh her mouth with vinegar, but this did not ſtop the diſcharge. In the afternoon I perceived a purple ſpot on her right cheek, and on removing her handkerchief ten or twelve more were diſcovered on her ſhoulders and breaſts, and a ſtill greater number on her right arm. Alarmed at theſe appearances, I requeſted the advice of a phyſician, who recommended a ſtrong decoction of bark and ſnake-root, with lemon juice, to be given as often as her ſtomach would bear it. At the ſame time ſhe was de-

ſired fired to eat plentifully of oranges, and to drink frequently of red port wine.

Blood continued to flow very faft from the mouth, and a little from the noftrils, which we filled with lint moiftened with *Aqua Styptica*. The mouth was frequently wafhed with the tincture of rofes, but the blood continuing to iffue from the gums, they were touched with aqua ftyptica.

*May* 3. This day the catamenia came on, and difcharged very confiderably. On enquiry we found it was a week before the ufual period. The patient's pulfe was ftrong and regular, and fhe did not feem to lofe her ftrength fo much as I expected, confidering the difcharge.

*May* 4. She had a natural ftool: the difcharge from the gums was fomewhat leffened; but the catamenia ftill continued in the fame degree as yefterday. We now perceived more fpots on her legs, and the next morning (after a very reftlefs night) we difcovered feveral frefh ones, particularly on one of her arms. Thofe that firft appeared were more florid than when we firft noticed them.

*May* 6. The difcharges were nearly the fame as before, but her pulfe was as good as if fhe had been in perfect health. At night fhe was extremely reftlefs.

May

*May* 7. The patient having had no evacuation by ſtool ſince Thurſday, a common glyſter was injected, and brought away with it a great quantity of black hardened fœces. She felt herſelf better after this evacuation and had a good night; but ſtill continued to ſpit a great deal of blood, and the catamenia did not abate.

*May* 8. She ſeemed to be rather better, when an hæmorrhage came on ſuddenly from the left noſtril. I found her pulſe much quickened, which I attributed to her hurry of ſpirits. She complained of ſickneſs, and vomited a pint baſon full of a dark coloured liquor mixed with a good deal of pure blood. The diſcharge from the noſe was ſtopped by ſtyptic applications to the noſtrils, but ſhe ſtill felt the blood flowing down her throat. She was now very pale and ſick, and vomited another pint baſon full of blood, &c. as before. The hæmorrhage, which began about twelve o'clock, abated about three, and at four o'clock entirely ceaſed.

*May* 9. She was much better. As ſhe had ſwallowed ſo much blood, and had not had a ſtool ſince the laſt glyſter, it was repeated, and with the ſame good effect as before. Before night the catamenia had quite ceaſed, the day following (Wedneſday, May 10) the ſaliva was diſ-

difcharged without the leaft tinge of blood. She now mended very rapidly.

*May* 11. I gave her ten grains of rhubarb with the decoction of bark, and repeated the fame dofe at night. This produced two natural ftools. Since that time fhe has taken the bark in fubftance, and milk from the cow night and morning; and is recovering her health and ftrength very faft. It is remarkable, that on the day after the bleeding from the nofe, the fpots that were before of a purple colour fuddenly changed to a bright red.

To inveftigate the caufe of this difeafe may be difficult, but I think it right to mention a circumftance which may perhaps throw fome light on it; and this is, that at home the patient feldom eats fifh, and it happened that during the week fhe fpent at her friends, they had frequent dinners and fuppers of fifh, of which fhe ate very heartily. Her gums too, fhe tells me, have for fome time paft been apt to bleed on the leaft touch.

To this cafe I fhall add another of a fimilar nature, but attended with much more formidable fymptoms, communicated to me by my brother-in-law, Mr. JOHN ROBINS, furgeon at Afhford, and which I fhall give in his own words, extracted tracted from his letter to me on the occasion. "In February 1780 Mrs. Sweetlove of Westwell, about four miles from Ashford, on waking in the morning perceived blood in her mouth, and by the time I saw her in the afternoon had spit into a bason a pint and a half of pure blood. Upon looking into her mouth I observed the blood streaming down the teeth of the upper jaw. I likewise perceived many extravasations in the mouth, and upon further examination discovered petechiæ in various parts of the body, and a sanies discharged by an issue and two ulcers. A day or two after this blood began to flow copiously from the vagina, so that in four days (before which the hæmorrhage did not seem to abate) she had lost a great quantity of blood. I gave her half a drachm of bark in substance every hour and a half, with mineral acids and tincture of roses, and directed her to eat Seville oranges and to drink red wine. On the second day of the uterine hæmorrhage a physician was sent for. He made no change in the mode of treatment, but we were soon obliged to vary it, for the acids disagreed that day with her stomach so much, that she kept nothing down, for which reason their use was from that time discontinued, and ten grains of

 ipeipecacoanha given as a puke before we began with the bark again. The patient continued the ufe of the bark till the latter end of May, when fhe tired of it, and in its ftead I gave her the *Cortex. Eleuther.* & *Vin. Amar.* Her diet confifted at firft almoft entirely of affes milk, but after fome little time fhe took animal food, and during the fummer months ate very largely of currants, which fhe was remarkably fond of. By thefe means before the end of Auguft, after experiencing a variety of fymptoms from a weak fibre and impoverifhed blood, fhe was reftored to a tolerable ftate of health. It feems right to add, that the patient is naturally of a highly fcorbutic habit with a fcrophulous taint, as is evident from feveral indurated glands about her neck and axillæ, and from a difpofition to hectic fever, which is probably owing to a fimilar caufe in the lungs."

*Sandwich*, May 18, 1781.

